# Supplementary material for: Stress‐induced host membrane remodeling protects from infection by non‐motile bacterial pathogens
Source: EMBO J. 2018 Nov 2;37(23):e98529. doi: 10.15252/embj.201798529 (PMC6276891; doi:10.15252/embj.201798529)
Supplement: Supplementary file 1 — Appendix [file EMBJ-37-e98529-s001.pdf]

‘Stress-induced host membrane remodeling protects from infection by non-motile bacterial pathogens’

Appendix contents

Supplementary Figures 2

Supplementary Figure Legends 5

Supplementary Materials and Methods 7

References for Supplementary Materials and Methods 9

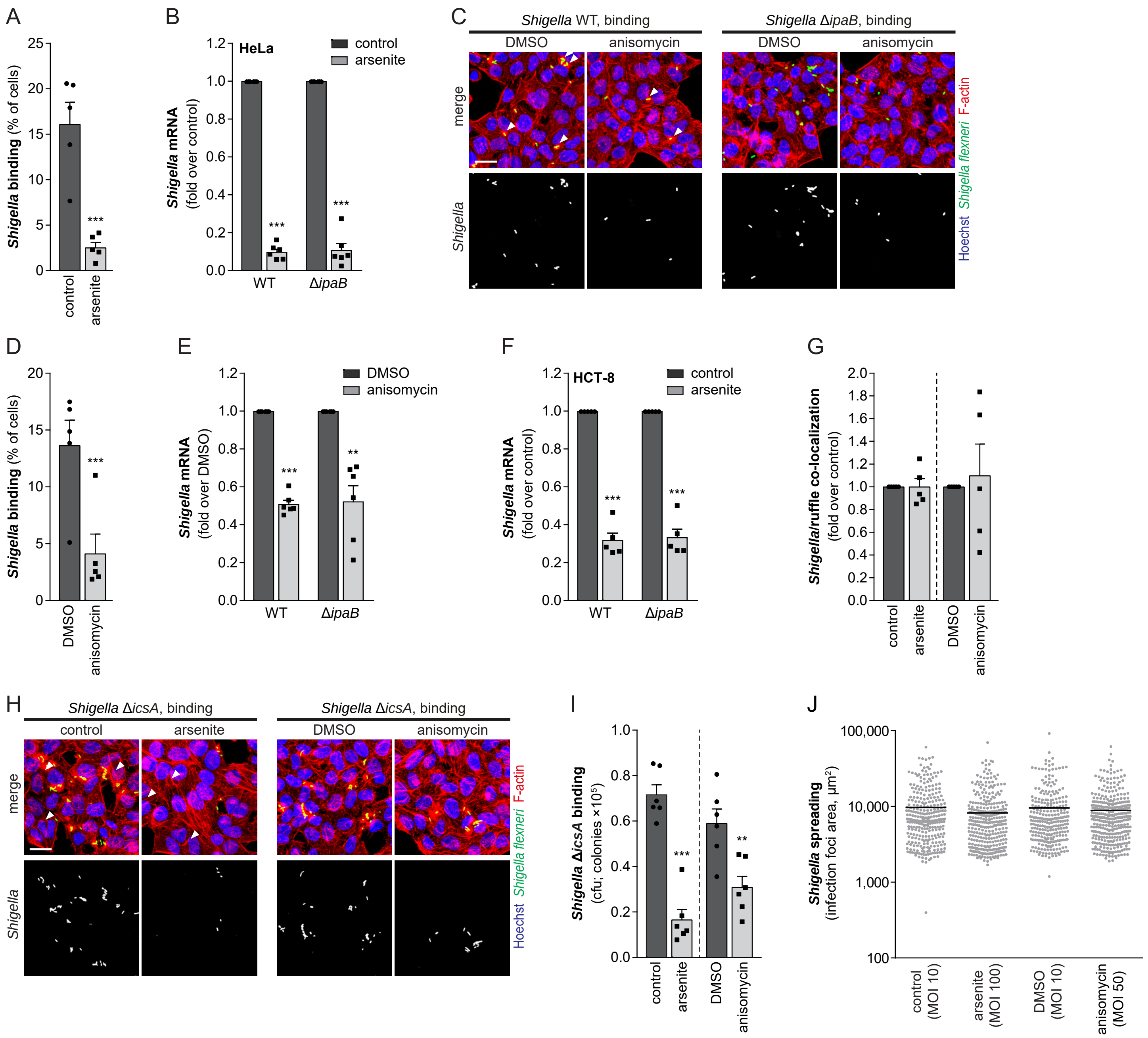
A
Shigella binding (% of cells)
control
arsenite
***
B
HeLa
control
arsenite
Shigella mRNA (fold over control)
WT
ΔipaB
***
C
Shigella WT, binding
Shigella ΔipaB, binding
DMSO
anisomycin
merge
Shigella
Hoechst Shigella flexneri F-actin
D
Shigella binding (% of cells)
DMSO
anisomycin
E
Shigella mRNA (fold over DMSO)
**
F
HCT-8
Shigella mRNA (fold over control)
G
Shigella/ruffle co-localization (fold over control)
H
Shigella ΔicsA, binding
I
Shigella ΔicsA binding (cfu; colonies ×10⁵)
J
Shigella spreading (infection foci area, µm²)
100,000
10,000
1,000
100
control (MOI 10)
arsenite (MOI 100)
DMSO (MOI 10)
anisomycin (MOI 50)

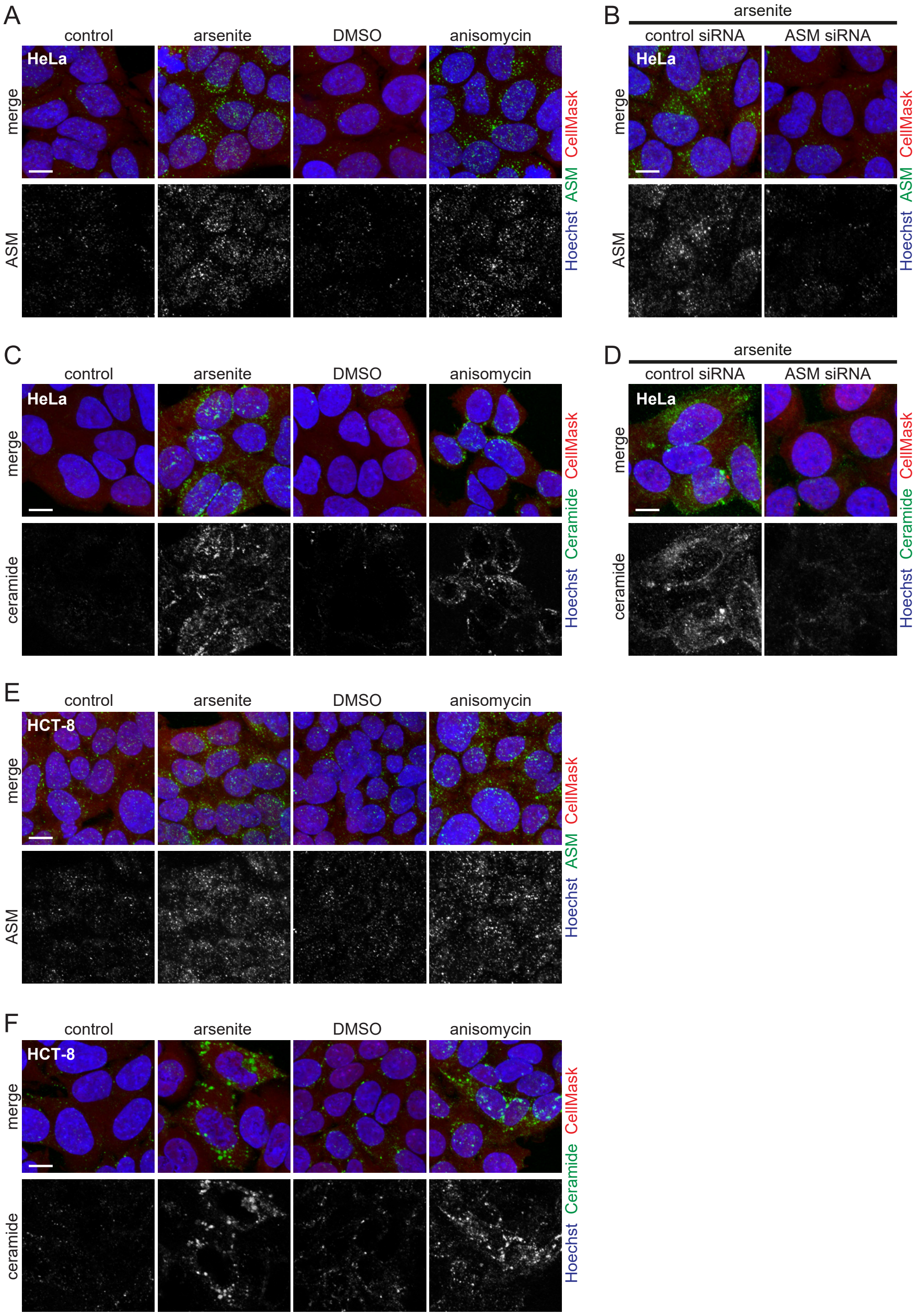
A
control
arsenite
DMSO
anisomycin
HeLa
merge
ASM
Hoechst ASM CellMask
B
arsenite
control siRNA
ASM siRNA
HeLa
merge
ASM
Hoechst ASM CellMask
C
control
arsenite
DMSO
anisomycin
HeLa
merge
ceramide
Hoechst Ceramide CellMask
D
arsenite
control siRNA
ASM siRNA
HeLa
merge
ceramide
Hoechst Ceramide CellMask
E
control
arsenite
DMSO
anisomycin
HCT-8
merge
ASM
Hoechst ASM CellMask
F
control
arsenite
DMSO
anisomycin
HCT-8
merge
ceramide
Hoechst Ceramide CellMask

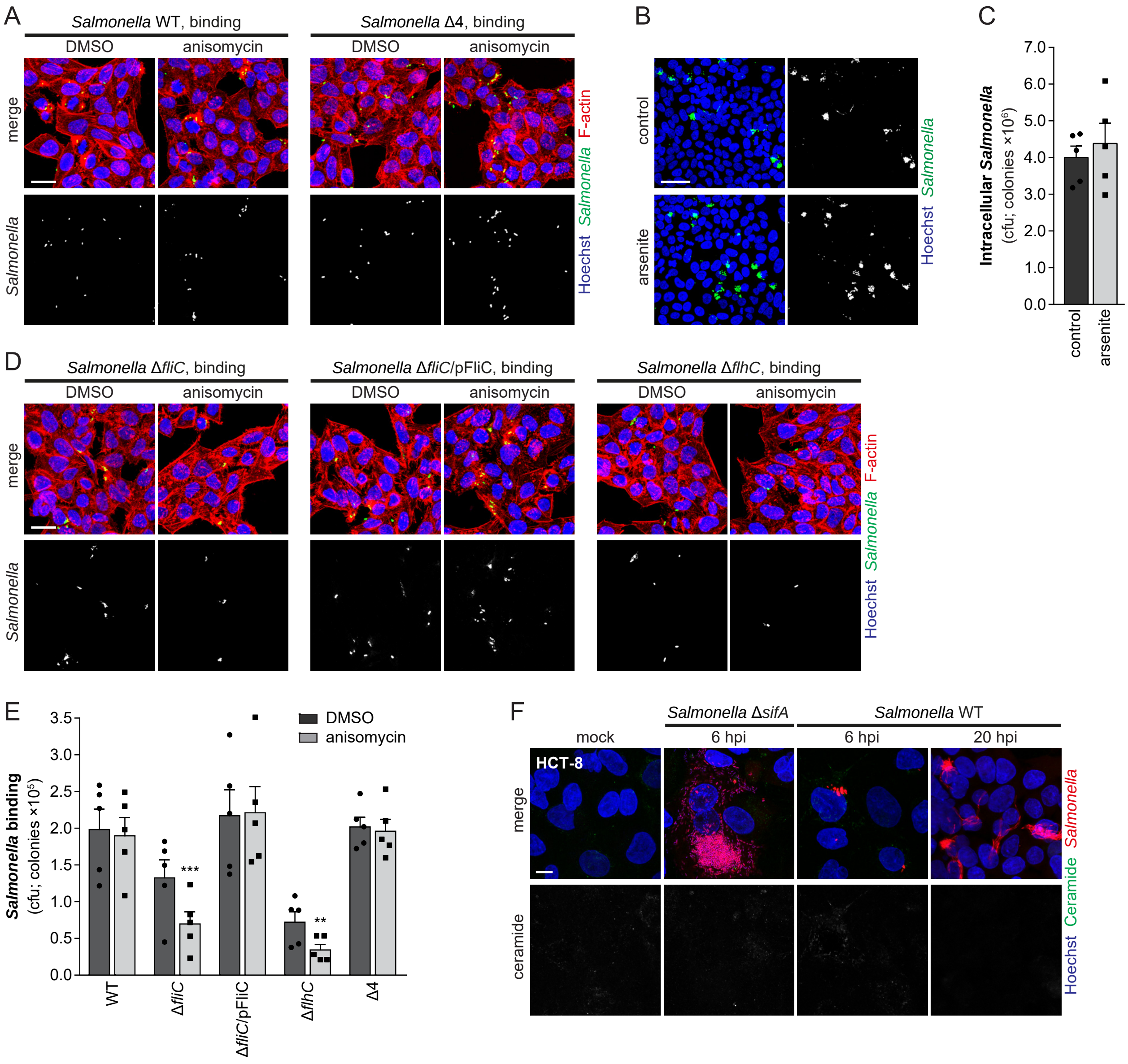
A
Salmonella WT, binding
Salmonella Δ4, binding
DMSO
anisomycin
merge
Salmonella
Hoechst Salmonella F-actin
B
control
arsenite
Hoechst Salmonella
C
Intracellular Salmonella
(cfu; colonies ×10^6)
7.0
6.0
5.0
4.0
3.0
2.0
1.0
0.0
control
arsenite
D
Salmonella ΔfliC, binding
Salmonella ΔfliC/pFliC, binding
Salmonella ΔflhC, binding
E
Salmonella binding
(cfu; colonies ×10^5)
3.5
3.0
2.5
2.0
1.5
1.0
0.5
0.0
DMSO
anisomycin
***
**
WT
ΔfliC
ΔfliC/pFliC
ΔflhC
Δ4
F
Salmonella ΔsifA
Salmonella WT
mock
6 hpi
20 hpi
HCT-8
merge
ceramide
Hoechst Ceramide Salmonella

Supplementary Figure Legends

Figure S1. Host cellular stress impairs *Shigella* binding to host cells, but not *Shigella* cell-to-cell spreading

A and D. Percentage of HeLa cells with bacteria bound after pre-treatment with arsenite (A), anisomycin (D), or corresponding controls, followed by incubation with *Shigella* WT for 25 min.

B. qRT-PCR quantification of bacteria bound to HeLa cells pre-treated or not with arsenite followed by incubation with *Shigella* WT or Δ*ipaB* mutant strain for 25 min.

C and E. Representative images (C) and qRT-PCR quantification (E) of bacteria bound to HeLa cells pre-treated with anisomycin or DMSO (control) followed by incubation with *Shigella* WT or Δ*ipaB* mutant strain for 25 min. Ruffle formation induced by *Shigella* WT in panel C is highlighted by white arrowheads.

F. qRT-PCR quantification of bacteria bound to HCT-8 cells pre-treated or not with arsenite followed by incubation with *Shigella* WT or Δ*ipaB* mutant strain for 25 min.

G. Percentage of ruffles induced upon bacterial contact to Hela cells pre-treated or not with arsenite or anisomycin followed by incubation with *Shigella* WT for 25 min.

H and I. Representative images (H) and cfu quantification (I) of bacteria bound to HeLa cells pre-treated with arsenite, anisomycin or corresponding controls followed by incubation with *Shigella* Δ*icsA* for 25 min.

J. Quantification of infection foci area of HeLa cells pre-treated with arsenite, anisomycin or corresponding controls and infected with *Shigella* WT, analyzed at 3 hpi.

Shigella infection was performed at MOI 10 for *Shigella* WT or MOI 50 for the Δ*ipaB* or Δ*icsA* mutant strains. Results are shown as mean ± s.e.m. from 5 (panels A, D, F, G, J) or 6 (panels B, E, I) independent experiments; *$p<0.05$, **$p<0.01$, ***$p<0.001$ (paired t-test). Scale-bar, 25 µm.

Figure S2. ASM is activated in response to cellular stress

A and C. Representative images of HeLa cells treated with arsenite or anisomycin and the corresponding controls, stained for ASM (A) or ceramide (C).

B and D. Representative images of HeLa cells transfected with ASM or control siRNA, treated with arsenite, stained for ASM (B) or ceramide (D).

E and F. Representative images of HCT-8 cells treated with arsenite or anisomycin and the corresponding controls, stained for ASM (E) or ceramide (F).

Scale bar, 10 µm.

Figure S3. Infection of non-motile *Salmonella* is inhibited upon host stress

A. Representative images of bacteria bound to HeLa cells pre-treated with anisomycin or DMSO (control) followed by incubation with *Salmonella* WT or Δ4 mutant strain.

B and C. Representative images (B) and cfu quantification (C) of Hela cells infected with *Salmonella* WT and treated with arsenite or control at 0.5 hpi (post-invasion) for 1 h. Analysis of infection was performed at 20 hpi.

D. Representative images of bacteria bound to HeLa cells pre-treated with anisomycin or DMSO followed by incubation with *Salmonella* Δ*fliC*, Δ*fliC* complemented strain (Δ*fliC*/pFliC) or Δ*flhC* mutant strains.

E. Cfu quantification of bacteria bound to HeLa cells pre-treated with anisomycin or DMSO followed by infection with *Salmonella* WT, Δ*fliC*, Δ*fliC*/pFliC, Δ*flhC* or Δ4.

F. Representative images of ceramide staining in HCT-8 cells infected with *Salmonella* WT or Δ*sifA* mutant strain, analyzed at the indicated times post-infection.

Salmonella infection was performed at MOI 50 and analyzed 10 min after bacterial incubation (panels A, D and E) or with MOI 25 and analyzed at 20 hpi (panels B and C). Results are shown as mean ± s.e.m. from 5 independent experiments; **p<0.01, ***p<0.001 (paired t-test for panel C; two-way ANOVA for panel E). Scale bar, 50 µm (panel B), 25 µm (panels A and D) and 10 µm (panel F).

Supplementary Materials and Methods

Cell Culture

Human epithelial HeLa-229 (HeLa CCL-2.1, ATCC) and Caco-2 (ATCC HTB-37) cells were cultured in DMEM GlutaMAX containing 1.0 g/l glucose (Life Technologies, 21885); human colon cancer HCT-8 (ATCC CCL-244) and HT-29 (ATCC HTB-38) cells were cultured in RPMI 1640 GlutaMAX (Life Technologies, 72400). Media were supplemented with 10% fetal bovine serum (Biochrom, S0615-1047D). Cells were maintained at 37°C in a 5% CO_2 humidified atmosphere. Unless otherwise specified, $6x10^4$ HeLa-229 or HCT-8 cells and $8x10^4$ HT-29 or Caco-2 cells were seeded in 24-well plates, 48 h before infection.

For the treatment of cells, all chemical compounds and the corresponding vehicles were added directly to the culture medium, prior to infection. The cells were incubated for the time periods indicated below, washed with PBS and subsequently infected. The following conditions were used: 0.1 mM sodium arsenite for 1 h (HeLa and HT-29) or 0.5 mM for 35 min (HCT-8 and Caco-2), 100 µg/ml puromycin for 1 h, 100 µM cycloheximide for 1 h, 10 µg/ml anisomycin for 20 min, 10 ng/ml TNF-α for 15 min, 5 µM SB203580 for 75 min, 50 µM amitriptyline for 75 min, 0.5 mM hydrogen peroxide for 1 h (HeLa) and 3 h (HCT-8), 0.5 µM GW4869 for 75 min and 10 mM N-acetyl-L-cysteine (NAC) for 1h. NAC, SB203580, amitriptyline or GW4869 were added in combination with the stressors (arsenite and anisomycin) for the overlapping incubation time. Similarly, NAC, amitriptyline or GW4869 were added in combination with 10 µg/ml of gentamycin in the re-infection experiments for the indicated times, prior to the secondary infection. For the hypoxia treatment, $1x10^5$ cells (HeLa) were seeded in 24-well plates and incubated for 24 h at 37°C in a 5% CO_2 humidified atmosphere. Subsequently, the cell medium was exchanged and the plates were incubated with no lid in an anaerobic jar containing an AnaeroGen pack (reducing the oxygen level in the jar to below 1%; Thermo Scientific Oxoid, AN0025A) for 15 h. The cells were re-oxygenated for 5-10 min prior to infection.

Bacterial Strains

Shigella flexneri serotype 5 strain M90T expressing GFP was obtained by transformation with the pXG-1 plasmid (Urban & Vogel, 2007) and is referred to as wild-type (WT) in this study. The *Shigella flexneri* strain expressing mCherry was obtained by transformation with either the pSB4004 (kindly provided by Prof. Jorge E. Galán, Yale University, USA) or pFPV-mCherry (Drecktrah et al, 2008) (kindly provided by Olivia Steele-Mortimer; Addgene #20956) plasmids. The *Shigella* isogenic Δ*icsA* and Δ*ipaB* mutant strains were previously described (Sunkavalli et al, 2017). The *Shigella* Δ*ipaB*/Invasin strain was obtained by transforming the ΔIpaB mutant strain with the pRI203 plasmid expressing the *Yersinia*

Invasin protein (Isberg et al, 1987); the pRI203 plasmid was kindly provided by Prof. Jorge E. Galán (Yale University, USA). *Salmonella enterica* serovar Typhimurium strain SL1344 expressing GFP constitutively from a chromosomal locus (Papenfort et al, 2009) and *Salmonella* expressing mCherry, obtained by transformation of pFPV-mCherry, were used in this study and referred to as wild-type (WT). The *Salmonella* Typhimurium SL1344 mutant strains Δ*fliC* and Δ4 strain (Δ*sopB*, Δ*sipA*, Δ*sopE*, Δ*sopE2*) were kindly provided by Dr. M. Kolbe (MPI-IB, Germany) and Prof. Jorge E. Galán (Yale University, USA), respectively. The *Salmonella enterica* serovar Typhimurium strain LT2 flhC5213::MudJ strain was kindly provided by Kelly T. Hughes (University of Utah, USA) and used as donor strain for P22 transduction of the *Salmonella enterica* serovar Typhimurium strain SL1344; the resulting strain is referred to as *Salmonella* Δ*flhC*. The *Salmonella* Δ*fliC* mutant strain was complemented with the protein FliC expressed from a plasmid encoding the *Salmonella fliC* ORF under the control of the endogenous promoter. The following primers were used for cloning the *fliC* ORF in the pXG-1 plasmid (replacing the *gfp* gene): 5'-CTTTGGACGTCTAGTTAAGCGCGTTATCGGC-3', and 5'-ACGTTTCTAGATTAACGCAGTAAAGAGAGGAC -3'.

Listeria monocytogenes serovar 1/2a EGD-e was used as WT. *Yersinia pseudotuberculosis* kindly provided by T. Ölschläger (IMIB, University of Würzburg, Germany) was transformed with the pXG-1 plasmid (Urban & Vogel, 2007) and was used as WT. *Shigella*, *Salmonella* and *Yersinia* were grown aerobically in Luria broth (LB) medium, and *Listeria* was grown in Brain Heart Infusion (BHI) medium. When appropriate, medium was supplemented with the following antibiotics: ampicillin 100 µg/ml, chloramphenicol 20 µg/ml, kanamycin 25 µg/ml.

For growth curve measurement, overnight bacterial cultures were diluted 1:100 and grown in 200 µl LB or LB with 0.1 mM sodium arsenite, DMSO or 10 µg/ml anisomycin for 15 h at 37˚C in 96-well plates, under continuous orbital shaking, and the OD_{600} was measured with the Tecan Infinite 200Pro plate reader, at 10 min intervals.

siRNA transfection

Reverse transfection of siRNAs into HeLa-229 cells was performed at a final concentration of 50 nM with Lipofectamine RNAiMAX (Life Technologies, 13778150), as described previously (Sunkavalli et al, 2017). For microscopy, cfu assay and RNA isolation, 6.0×10^5 HeLa-229 cells were seeded in 24-well plates. Medium was exchanged 24 h prior to infection, which was performed as described above at 62 h post-transfection. siGENOME non-targeting siRNA #5 (D-001210-05), siGENOME Human MAPK14 SMARTpool (M-003512-06) and siGENOME Human SMPD1 SMARTpool (M-006676-01) were purchased from Dharmacon, GE Healthcare.

RNA isolation and quantitative real-time PCR

For total RNA isolation, cells were directly lysed in TRIzol (Life Technologies) and extracted by phenol-chloroform followed by isopropanol precipitation. Total RNA was reverse-transcribed using hexameric random primers (Life Technologies) and M-MLV reverse transcriptase (Life Technologies), according to the manufacturer's instructions. Real-time quantitative analysis was performed using SsoAdvanced Universal SYBR Green Supermix (BioRad) according to the manufacturer's instructions, using a CFX96 TouchTM Real-Time PCR detection system (BioRad). The following primer pairs were used: GFP 5'-ATGCTTTTCCCGTTATCCGG-3' and 5'-GCGTCTTGTAGTTCCCGTCATC-3'; mCherry 5' GGCGAGTTCATCTACAAG 3' and 5' GGTCTTCTTCTGCATTACG 3'; human *smpd1* (ASM) 5' GCTGGAATTATTACCGAAT 3' and 5'TCATCATAGAAGACCTCAA 3'; β-actin 5'-CCTGTACGCCAACACAGTGC-3' and 5'-ATACTCCTGCTTGCTGATCC-3'. Expression was normalized to β-actin and the $2^{-\Delta\Delta Ct}$ method was used to calculate fold changes.

Protein extracts and Western-blot

Cells were washed in PBS, collected in Laemmli's sample buffer and separated on 10% SDS-PAGE, followed by Western-blotting. The following antibodies were used: β-actin (1:3,000; Sigma, A2228), Phospho-p38 MAPK (1:2,000; Cell Signaling Technology, 9216),p38 MAPK (1:1,000; Cell Signaling Technology, 9212) and human acid sphingomyelinase (1:200; Santacruz, sc-293189 (4H2)). Anti-mouse or anti-rabbit secondary antibody coupled to horseradish peroxidase were used (1:10,000; GE Healthcare, NA931 (anti-mouse) and NA934 (anti-rabbit)). Signals were detected using SuperSignal West Dura Extended Duration Substrate (Pierce, 34075) in an ImageQuant LAS 4000 CCD imaging system (GE Healthcare).

References for Supplementary Materials and Methods

Drecktrah D, Levine-Wilkinson S, Dam T, Winfree S, Knodler LA, Schroer TA, Steele-Mortimer O (2008) Dynamic behavior of Salmonella-induced membrane tubules in epithelial cells. *Traffic* **9:** 2117-2129

Isberg RR, Voorhis DL, Falkow S (1987) Identification of invasin: a protein that allows enteric bacteria to penetrate cultured mammalian cells. *Cell* **50:** 769-778

Papenfort K, Said N, Welsink T, Lucchini S, Hinton JC, Vogel J (2009) Specific and pleiotropic patterns of mRNA regulation by ArcZ, a conserved, Hfq-dependent small RNA. *Molecular microbiology* **74:** 139-158

Sunkavalli U, Aguilar C, Silva RJ, Sharan M, Cruz AR, Tawk C, Maudet C, Mano M, Eulalio A (2017) Analysis of host microRNA function uncovers a role for miR-29b-2-5p in Shigella capture by filopodia. *PLoS pathogens* **13:** e1006327

Urban JH, Vogel J (2007) Translational control and target recognition by Escherichia coli small RNAs in vivo. *Nucleic acids research* **35:** 1018-1037
