# Supplementary material for: Stress‐induced host membrane remodeling protects from infection by non‐motile bacterial pathogens
Source: EMBO J. 2018 Nov 2;37(23):e98529. doi: 10.15252/embj.201798529 (PMC6276891; doi:10.15252/embj.201798529)
Supplement: Supplementary file 2 — Expanded View Figures PDF [file EMBJ-37-e98529-s002.pdf]

Expanded View Figures

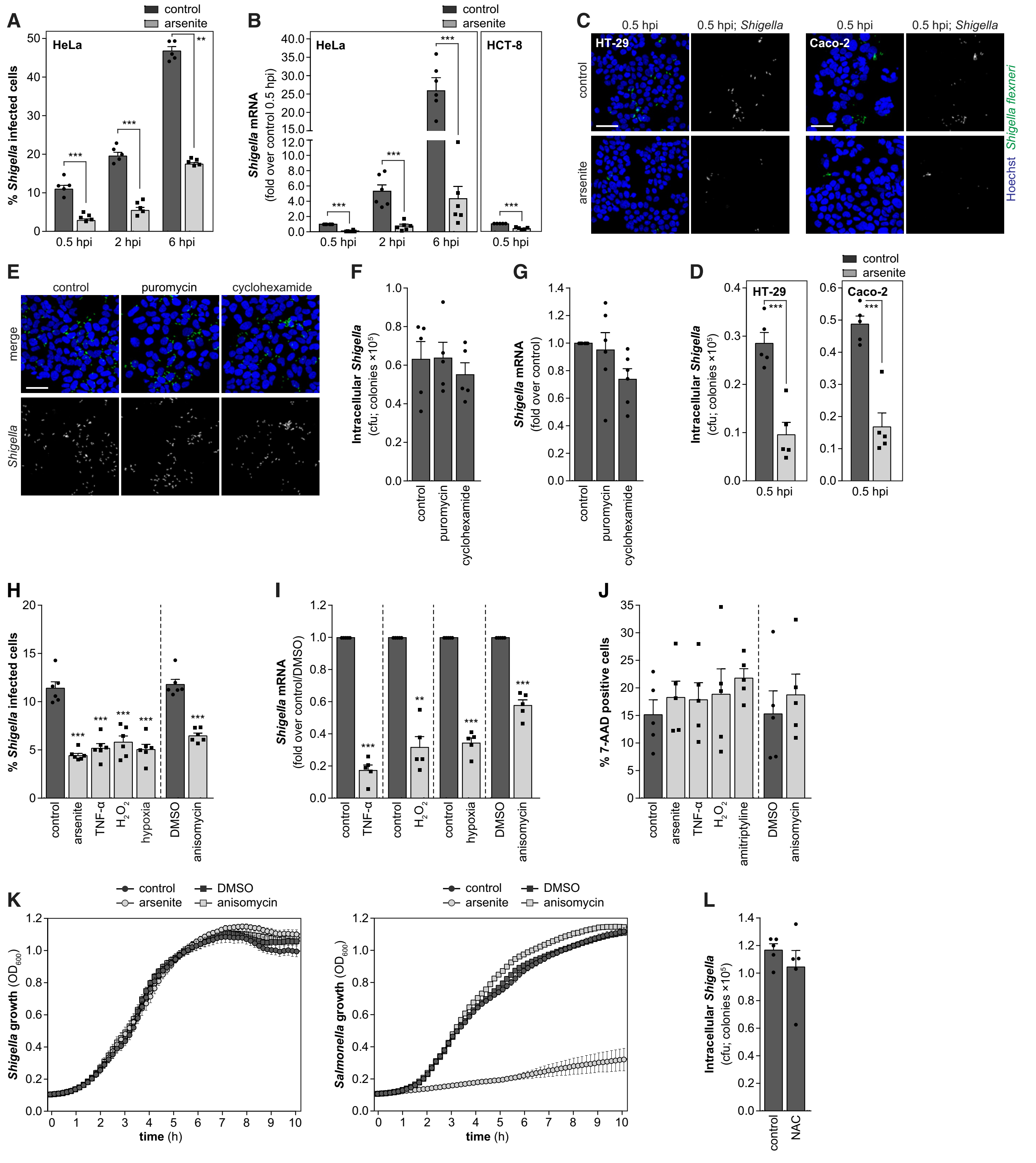

Figure EV1.

 © 2018 The Authors

Figure EV1. *Shigella* infection is inhibited by host cell stress.

A Percentage of HeLa cells infected with *Shigella* after pre-treatment with arsenite or control, analyzed at 0.5, 2, and 6 hpi.
B qRT–PCR quantification of intracellular bacteria in HeLa and HCT-8 cells pre-treated or not with arsenite and infected with *Shigella* WT. Analysis was performed at 0.5, 2, and 6 hpi for HeLa cells and at 0.5 hpi for HCT-8 cells. Results are shown normalized to the control at 0.5 hpi.
C, D Representative images (C) and cfu quantification (D) of HT-29 or Caco-2 cells pre-treated or not with arsenite and infected with *Shigella* WT, analyzed at 0.5 hpi.
E–G Representative images (E), cfu (F), and qRT–PCR (G) quantification of intracellular bacteria in HeLa cells pre-treated with puromycin or cycloheximide, or control, and infected with *Shigella*. Analysis was performed at 0.5 hpi.
H Percentage of HeLa cells infected with *Shigella* WT after pre-treatment with TNF-α, H_2O_2, anisomycin, hypoxia, and corresponding controls, analyzed at 0.5 hpi.
I qRT–PCR quantification of intracellular bacteria in HeLa cells infected with *Shigella* WT after pre-treatment with TNF-α, H_2O_2, anisomycin, hypoxia, and corresponding controls, analyzed at 0.5 hpi.
J Percentage of 7-AAD-positive cells following treatment with arsenite, TNF-α, H_2O_2, amitriptyline, anisomycin, and corresponding controls.
K Growth curve of *Shigella* WT or *Salmonella* WT (OD_{600}) in LB medium (10 h) in the presence of arsenite, anisomycin, or corresponding controls.
L Cfu quantification of intracellular bacteria in HeLa cells treated or not with NAC and infected with *Shigella* WT, analyzed at 0.5 hpi.

Data information: *Shigella* infection was performed at MOI 10. Results are shown as mean ± s.e.m. of 5 (panels A, B—HCT-8, D, F, I, J, K, L) or 6 (panels B—HeLa, G, H) independent experiments; $^{**}P < 0.01$, $^{***}P < 0.001$ (*t*-test adjusted for multiple comparison for panels A and B—HeLa; paired *t*-test for panels B—HCT-8, D, I, and L; one-way ANOVA for panels F–H and J). Scale bars, 50 μm.

Figure EV2. Neutral sphingomyelinase (NSM) inhibition does not affect *Shigella* infection upon host cell stress.

A–C Representative images (A), cfu (B), and qRT–PCR (C) quantification of intracellular *Shigella* in HeLa cells, pre-treated with arsenite, with arsenite plus the NSM inhibitor GW4869, or control.
D–F Representative images (D), cfu (E), and qRT–PCR (F) quantification of intracellular *Shigella* in HeLa cells, pre-treated with anisomycin, with anisomycin plus GW4869, or DMSO (control).
G, H Cfu (G) and qRT–PCR (H) quantification of intracellular bacteria in HeLa cells treated or not with amitriptyline and infected with *Shigella* WT.
I, J Cfu (I) and qRT–PCR (J) quantification of intracellular bacteria in HeLa cells transfected with ASM or control siRNA.
K ASM enzymatic activity quantification in HeLa cells, treated with arsenite, with arsenite plus the ASM inhibitor amitriptyline, or control. The ASM enzymatic activity was determined in the membrane fraction corresponding to 3.0×10^5 cells per condition.
L, M Ceramide quantification in HeLa cells, treated with arsenite (L) or anisomycin (M), with the stressors plus amitriptyline, or control. Ceramide levels are shown normalized to mock-treated cells.
N Analysis of *smpd1* (ASM) expression determined by qRT–PCR in HeLa cells transfected with ASM or control siRNA. Results are shown normalized to cells transfected with control siRNA.
O Western blot analysis of ASM levels in HeLa cells transfected with ASM siRNA or control siRNA; β-actin was used as loading control.
P ASM enzymatic activity quantification in HeLa cells transfected with ASM siRNA or control siRNA, and treated or not with arsenite. The ASM enzymatic activity was determined in the membrane fraction corresponding to 3.0×10^5 cells per condition.

Data information: *Shigella* infection was performed at MOI 10 and analyzed at 0.5 hpi. Results are shown as mean ± s.e.m. of 5 (panels J, K–M, and P), 6 (panel I), or 7 (panels B, C, E–H, and N) independent experiments; $^{*}P < 0.05$, $^{**}P < 0.01$, $^{***}P < 0.001$ (one-way ANOVA for panels B, C, E, F, K–M; paired *t*-test for panels G–J and N; two-way ANOVA for panel P). Scale bars, 50 μm.
Source data are available online for this figure.

© 2018 The Authors

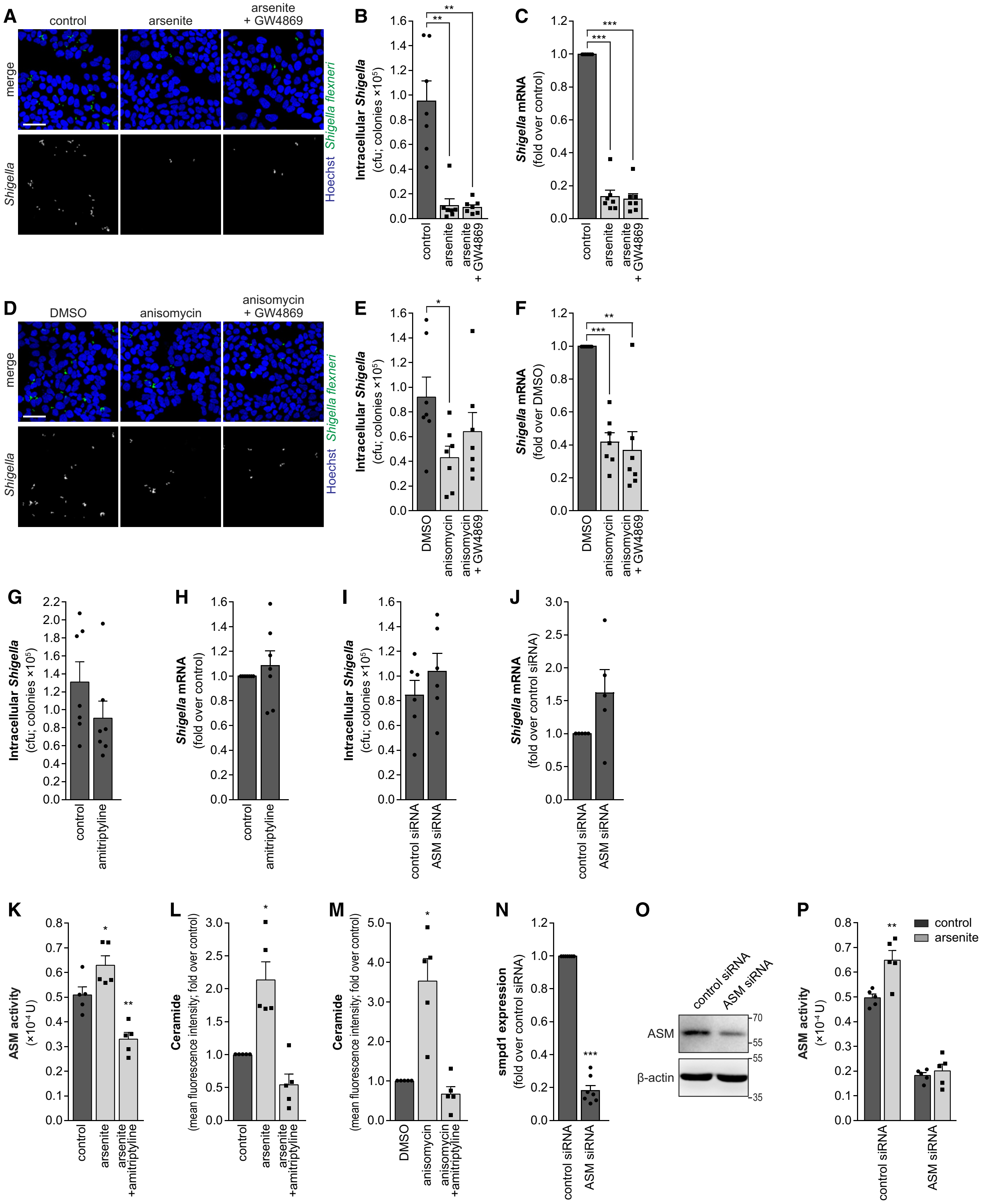

Figure EV2.

 © 2018 The Authors

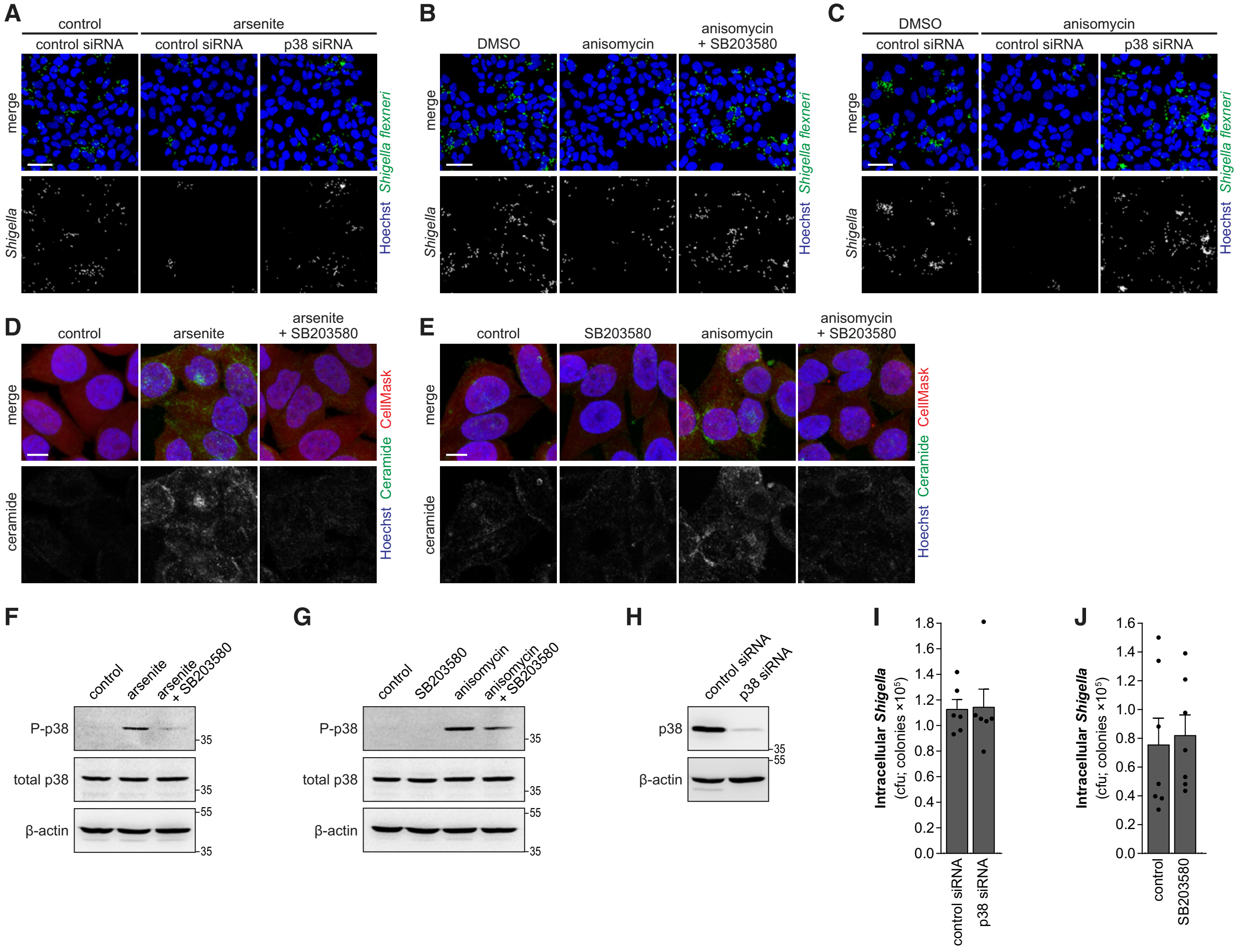

Figure EV3. MAPK p38 inhibition restores *Shigella* infection of host cells exposed to stress.

A–C (A and C) Representative images of *Shigella* infection in HeLa cells transfected with p38 siRNA or control siRNA and pre-treated or not with arsenite (A) or anisomycin (C) prior to infection. (B) Representative images of *Shigella* infection in HeLa cells pre-treated with anisomycin, with anisomycin plus SB203580, or control.

D, E Representative images of HeLa cells treated with arsenite (D) or anisomycin (E) and the corresponding controls in the presence or not of SB203580, stained for ceramide.

F, G Western blot analysis of p38 MAPK phosphorylation in HeLa cells treated with arsenite (F) or anisomycin (G) in the presence or not of SB203580. Phosphorylated and total p38 proteins are shown; β-actin was used as loading control.

H Western blot analysis of p38 levels in HeLa cells transfected with p38 siRNA or control siRNA; β-actin was used as loading control.

I Cfu quantification of intracellular bacteria in HeLa cells transfected with p38 or control siRNA.

J Cfu quantification of intracellular bacteria in HeLa cells treated or not with SB203580 and infected with *Shigella* WT.

Data information: *Shigella* infection was performed at MOI 10 and analyzed at 0.5 hpi. Results are shown as mean ± s.e.m. of 6 (panel I) or 7 (panel J) independent experiments; Scale bars, 50 μm (panels A–C) and 10 μm (panels D and E).

Source data are available online for this figure.

© 2018 The Authors

Figure EV4. ***Shigella*** **intracellular replication inhibits re-infection by non-motile bacteria.**

A, B qRT–PCR quantification of the secondary infection with *Shigella* WT (A) or *Salmonella* WT (B) of HeLa cells primarily infected with *Shigella* WT or Δ*ipaB*/Inv mutant strain, or mock-treated. Results are shown normalized to mock-treated cells.

C, D Cfu (C) and qRT–PCR (D) quantification of the primary infection by *Shigella* WT or Δ*ipaB*/Inv mutant strain of HeLa cells (corresponding secondary infection shown in Fig 5B–E).

E–J (E and H) Representative images of re-infection assays with *Shigella* WT (E) or *Salmonella* WT (H) of HCT-8 cells primarily infected with *Shigella* WT or Δ*ipaB*/Inv mutant strain, or mock-treated, as depicted in Fig 5A. Top panels show merge image with primary infection (red, mCherry-expressing *Shigella* strains) and secondary infection (green, GFP-expressing bacteria); bottom panels show secondary infection exclusively. (F, G, I, and J) Cfu (F and I) and qRT–PCR (G and J) quantification of the secondary infection with *Shigella* WT (F and G) or *Salmonella* WT (I and J) of HCT-8 cells primarily infected with *Shigella* WT or Δ*ipaB*/Inv mutant strain, or mock-treated.

K, L Cfu (K) and qRT–PCR (L) quantification of the primary infection by *Shigella* WT or Δ*ipaB*/Inv mutant strain of HCT-8 cells (corresponding secondary infection shown in Fig EV4E–J).

M Representative images of the re-infection assays with *Shigella* WT in HeLa cells primarily infected with *Salmonella* WT or mock-treated. Top panels show merge image with primary infection (red, *Salmonella* WT) and secondary infection (green, *Shigella*); secondary infection is shown in bottom panels.

N Cfu quantification of the secondary infection with *Shigella* in HeLa cells primarily infected with *Salmonella* WT or mock-treated.

O Cfu quantification of the primary infection by *Shigella* WT in HeLa cells re-infected with *Salmonella* Δ*fliC*, Δ*fliC*/pFliC, or Δ*flhC* mutant strains.

Data information: Infection was performed at MOI 10 for *Shigella* WT or MOI 350 for *Shigella* Δ*ipaB*/Inv and MOI 25 for *Salmonella* WT or MOI 50 for the *Salmonella* Δ*fliC*, Δ*fliC*/pFliC, or Δ*flhC* mutant strains. Results are shown as mean ± s.e.m. of five independent experiments; *$P < 0.05$, ***$P < 0.001$ (paired *t*-test for panel N; one-way ANOVA for all others). Scale bars, 50 μm.

 © 2018 The Authors

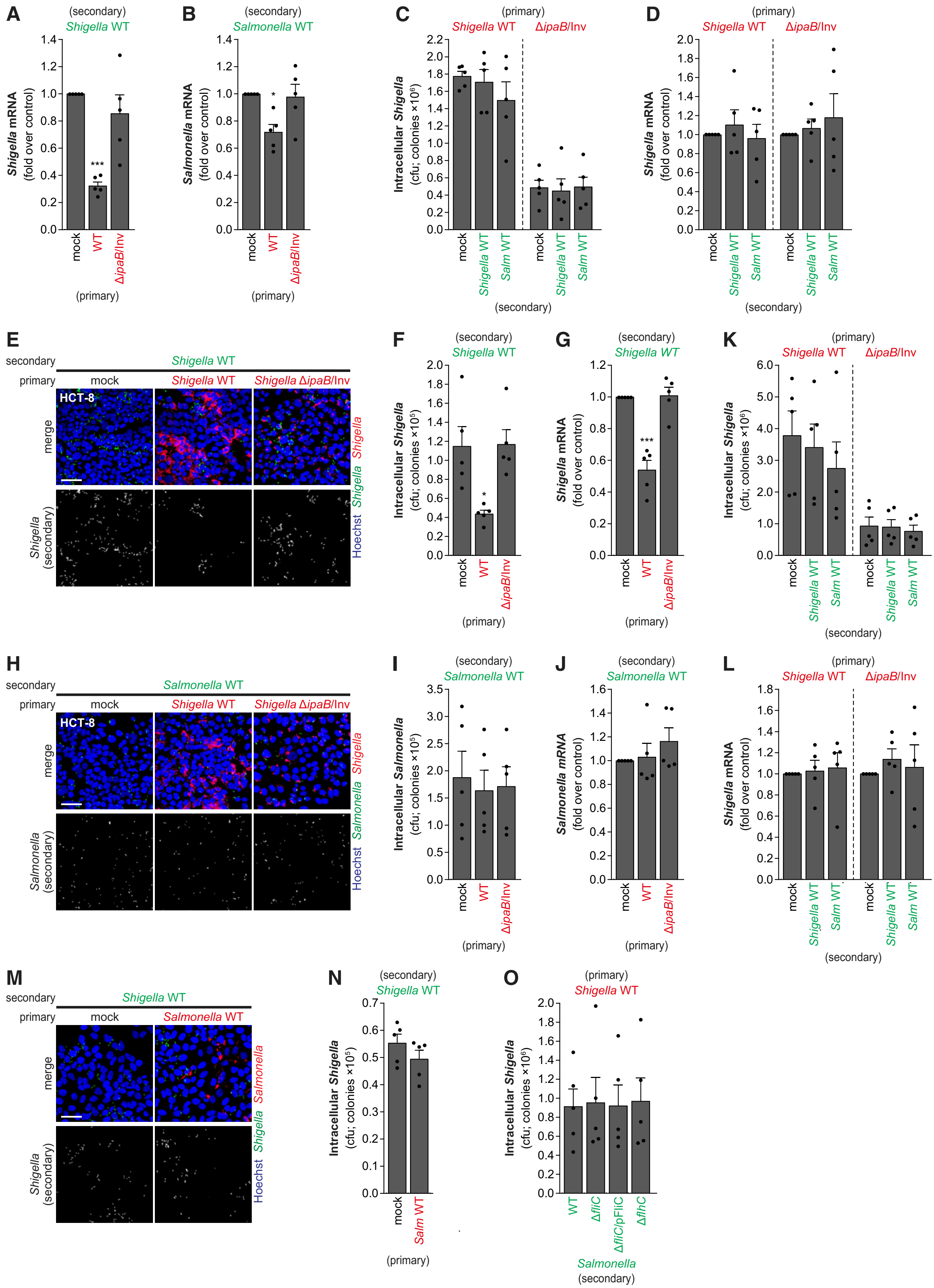
A
(secondary)
Shigella WT
Shigella mRNA (fold over control)
mock
WT
ΔipaB/Inv
(primary)
B
(secondary)
Salmonella WT
Salmonella mRNA (fold over control)
(primary)
C
(primary)
Shigella WT
ΔipaB/Inv
Intracellular Shigella (cfu; colonies ×10⁶)
Shigella WT
Salm WT
(secondary)
D
(primary)
Shigella mRNA (fold over control)
E
secondary
primary
HCT-8
merge
Shigella (secondary)
Hoechst
F
Intracellular Shigella (cfu; colonies ×10⁵)
G
K
H
Salmonella (secondary)
I
Intracellular Salmonella (cfu; colonies ×10⁵)
J
L
M
N
O
ΔfliC
ΔfliC/pFliC
ΔflhC
Salmonella

Figure EV4.

Figure EV5. ***Shigella*** **replication induces ASM activation.**

A–F (A, D, E, and F) Representative images of re-infection assays with *Shigella* WT of HeLa cells primarily infected with *Shigella* WT or mock-treated, following amitriptyline (A), p38 knockdown with siRNA (D), SB203580 (E), or NAC (F). Top panels show merge image with primary infection (red, mCherry) and secondary infection (green, GFP); secondary infection is shown in bottom panels. (B and C) qRT–PCR quantification of the secondary infection with *Shigella* WT, following the ASM siRNA (B) or amitriptyline (C) treatment as described in Fig 6H.

G Cfu quantification of the secondary infection with *Shigella* WT following NAC treatment.

H–N Cfu (H, J, L–N) and qRT–PCR (I, K) quantification of the primary infection by *Shigella* WT in HeLa cells (corresponding secondary infection shown in Figs 6I–M and EV5A–G).

O–S Representative images of ASM (O and R) or ceramide (P, Q, and S) staining in HeLa cells infected with *Shigella* WT (O, P, and Q) or Δ*ipaB*/Inv mutant strain (R and S), analyzed at 3 hpi.

Data information: *Shigella* infection was performed at MOI 10 (panels A–N), MOI 100 (panels O–Q), or MOI 350 (panels R and S). Results are shown as mean ± s.e.m. of 5 (panels B, C, G, I, J, L–N) or 6 (panels H and K) independent experiments; *$P < 0.05$ (one-way ANOVA for panels B, C, and G; paired *t*-test for all others). Scale bars, 50 μm (panels A, D–F), 10 μm (panels O, P, R, and S), and 25 μm (panel Q).

© 2018 The Authors

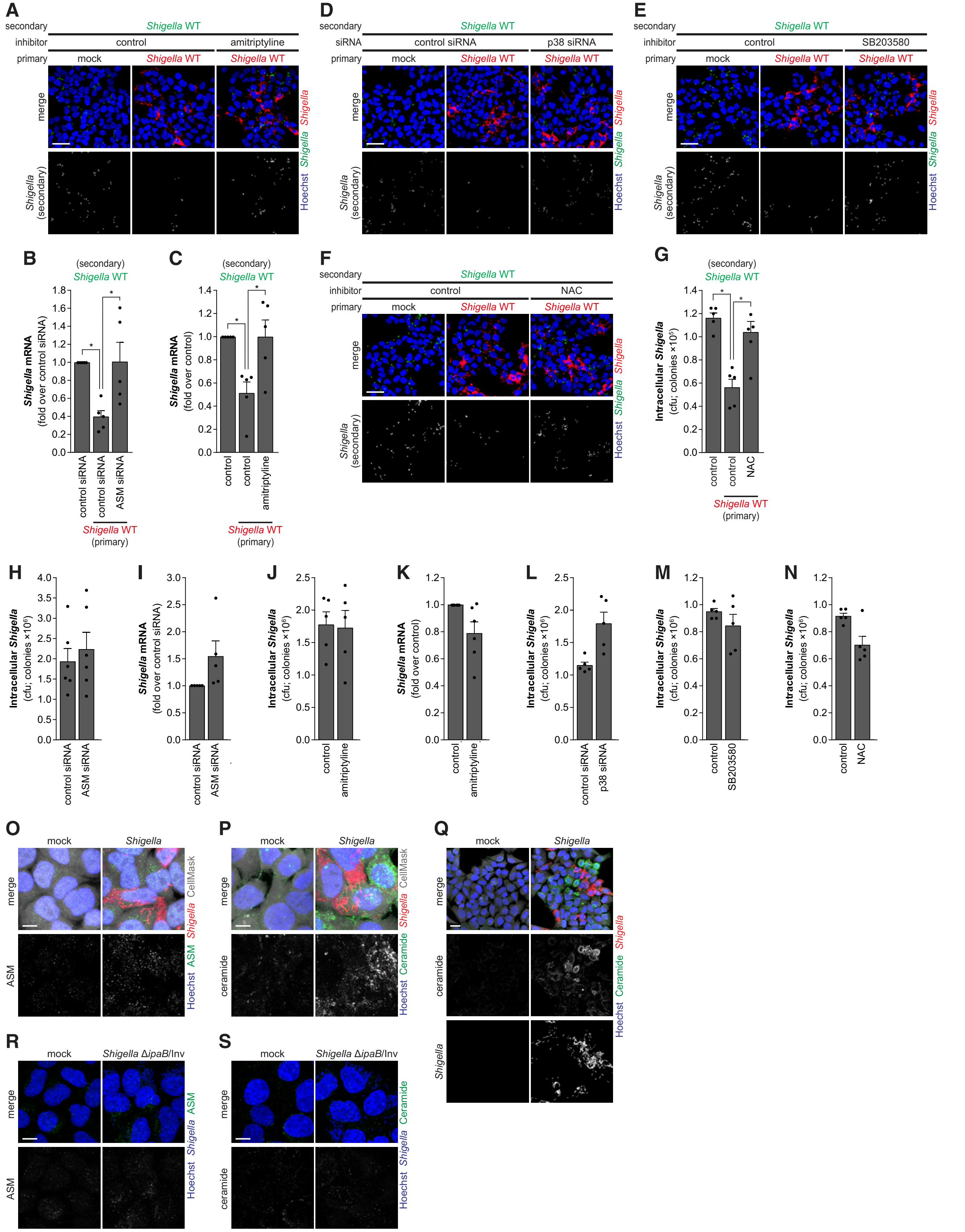
A
secondary
Shigella WT
inhibitor
control
amitriptyline
primary
mock
Shigella WT
merge
Shigella (secondary)
Shigella Shigella Hoechst
B
(secondary) Shigella WT
Shigella mRNA (fold over control siRNA)
control siRNA
ASM siRNA
Shigella WT (primary)
C
Shigella mRNA (fold over control)
control
amitriptyline
D
siRNA
control siRNA
p38 siRNA
E
SB203580
F
NAC
G
Intracellular Shigella (cfu; colonies ×10^5)
H
Intracellular Shigella (cfu; colonies ×10^6)
I
J
K
L
M
N
O
mock
Shigella
ASM
Hoechst ASM Shigella CellMask
P
ceramide
Hoechst Ceramide Shigella CellMask
Q
Hoechst Ceramide Shigella
R
Shigella ΔipaB/Inv
Hoechst Shigella ASM
S
Hoechst Shigella Ceramide

Figure EV5.
