# Supplementary figures and images for: Stress‐induced host membrane remodeling protects from infection by non‐motile bacterial pathogens
Source: EMBO J. 2018 Nov 2;37(23):e98529. doi: 10.15252/embj.201798529 (PMC6276891; doi:10.15252/embj.201798529)

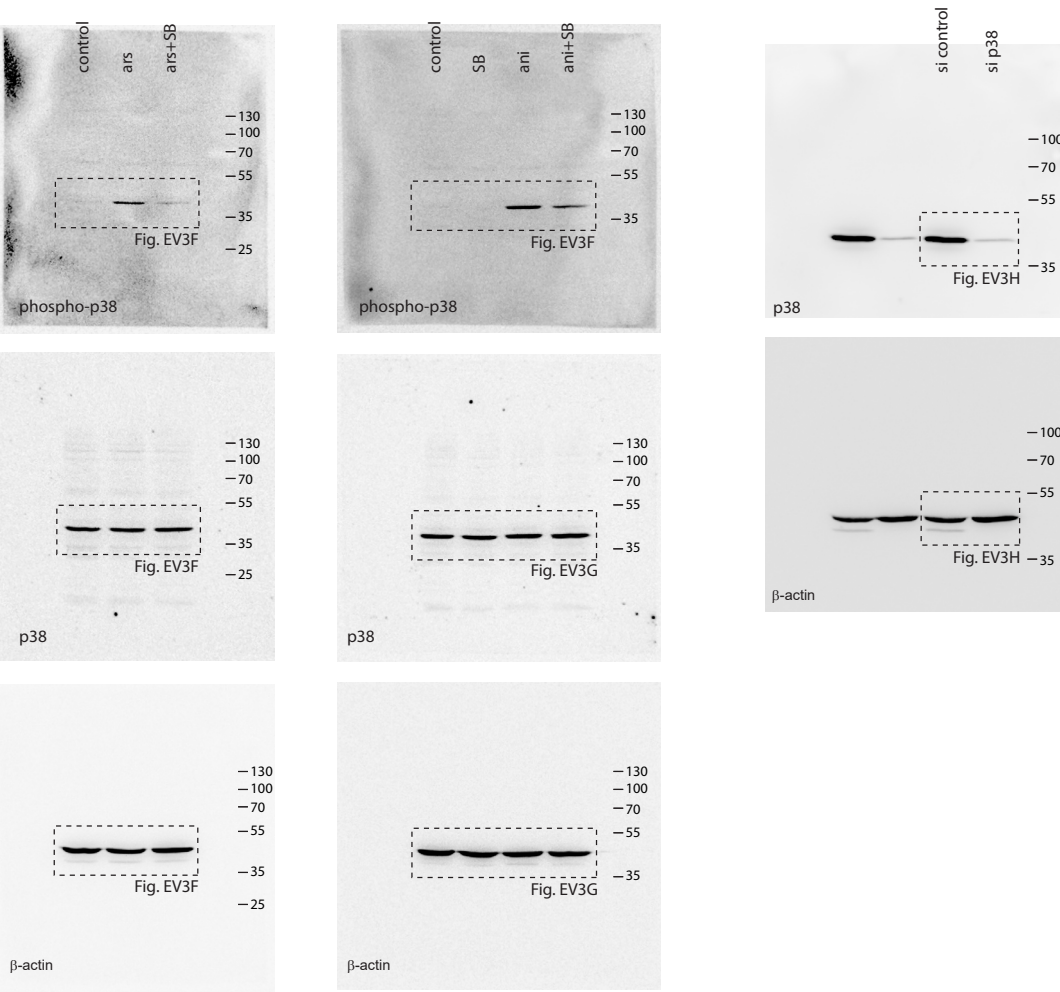

Supplement: Supplementary file 3 — Source Data for Expanded View [file EMBJ-37-e98529-s004.zip › embj201798529-sup-0005-SDataFigEV3.pdf]

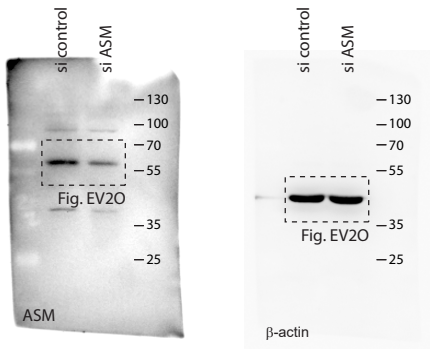

Supplement: Supplementary file 3 — Source Data for Expanded View [file EMBJ-37-e98529-s004.zip › embj201798529-sup-0004-SDataFigEV2.pdf]

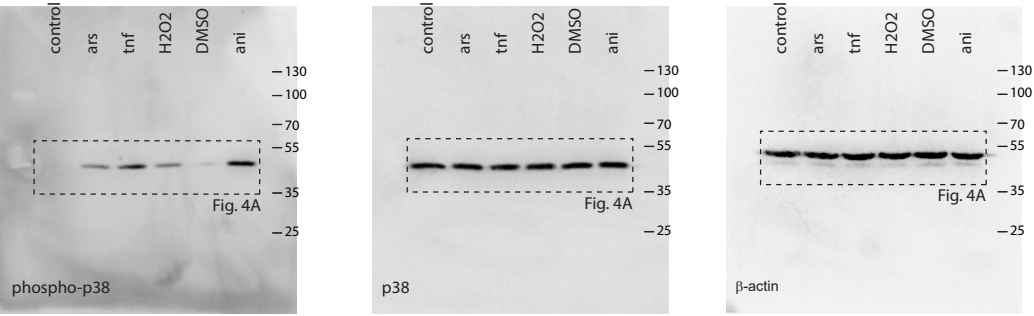

Supplement: Supplementary file 5 — Source Data for Figure 4 [file EMBJ-37-e98529-s003.pdf]
